# Human Papillomavirus Typing in HIV-Positive Women

**DOI:** 10.1155/S1064744901000229

**Published:** 2001

**Authors:** Sebastian Faro

**Affiliations:** Department of Obstetrics, Gynecology, and Reproductive Sciences The University of Texas – Houston Health Science Center , and The Woman's Hospital of Texas 7600 Fannin Houston TX 77054 USA


Infect Dis Obstet Gynecol 2001;9:123

### Comment: Human papillomavirus typing in HIV-positive women

**Sebastian Faro**

*Department of Obstetrics, Gynecology, and Reproductive Sciences, The University of Texas – Houston Health Science Center, and The Woman's Hospital of Texas, Houston, TX*

In this paper (page 89 of this issue), Hameed and colleagues set out to study the presence of low- and high-risk human papillomavirus (HPV) types in women infected by human immunodeficiency virus (HIV), and to correlate the presence of HPV with Pap smear results.

It is not surprising to find HPV to be present in the cervices of HIV-infected women even though their Pap smears were not abnormal. What was interesting, but also not surprising, was the finding that low-risk HPV types, as well as high-risk types, were present in the absence of significant cervical disease (Table 1 in their paper). It is a well-known fact that the Pap smear is not a particularly sensitive method for detecting HPV.

The authors found 47% of the HIV-infected women to harbor HPV in their cervices. They also found that 80% of HPV-positive patients who had Pap smears showing benign cellular change or atypical squamous cells of undetermined significance (ASCUS) had high-risk HPV types. However, in the face of a relative absence of significant cervical disease, such as moderate or severe dysplasia, the authors recommend routine HPV typing.

However, the authors do not discuss the fact that in this study there was a significant absence of cervical dysplasia although high-risk HPV types were present. They do not discuss the spontaneous remission rate or the progress rate. The authors do not make any recommendations with regard to management of patients with high-risk HPV types, appropriate follow-up, treatment regimens, and whether or not such HPV typing programs would be cost-effective.

Correspondence to: Sebastian Faro, MD, PhD, Department of Obstetrics, Gynecology, and Reproductive Sciences, The Woman's Hospital of Texas, 7600 Fannin, Houston, TX 77054

